# A Case of Basilar Artery Thrombus Improved With Anticoagulation

**DOI:** 10.7759/cureus.47079

**Published:** 2023-10-15

**Authors:** Divya Rajasekaran, Dilip Jayaraman

**Affiliations:** 1 Medicine, Sri Ramaswamy Memorial (SRM) Medical College Hospital and Research Center, Chennai, IND; 2 Neurology, Main Line Health, Paoli, USA

**Keywords:** vertebrobasilar stroke, neurointervention, acute cardioembolic stroke, basilar artery occlusion, stroke

## Abstract

Basilar artery (BA) occlusions are rare causes of ischemic strokes but can lead to devastating consequences if not addressed immediately. Herein, we discuss a case of an ischemic stroke due to near occlusion of the basilar artery with a good outcome due to timely presentation and intervention.

## Introduction

In 2019, stroke was the second leading cause of death globally, accounting for about 11.6% of all deaths [1]. Stroke is also a leading cause of long-term disability leading to significant impairments in a patient's quality of life.

Efforts are made throughout the world to increase awareness of stroke and encourage early medical attention. If left untreated, it is estimated that the patient loses about 1.9 million neurons every minute [2].

The basilar artery (BA) is a major blood vessel that supplies posterior circulation territories. Clinical presentation varies depending on the location and can sometimes lead to devastating consequences if left untreated. If patients present within the approved thrombolytic window, they should receive IV thrombolytics. If the vessel imaging shows basilar artery occlusion, the patient could benefit from mechanical intervention and thrombectomy [3]. Further workup should be done to identify the causative factors and appropriate risk modification.

## Case presentation

A 66-year-old right-handed Caucasian male with a past medical history of hypertension on metoprolol 50 mg extended release once daily and prior tobacco use disorder came to the hospital to be evaluated for vertigo, which started on the day of arrival. Symptoms began with headaches and occipital pain, which felt like muscle strain. Subsequently, he vomited for 20-30 minutes, followed by acute onset of vertiginous symptoms. The symptoms resolved entirely in about 30 minutes. The baseline modified Rankin scale was 0 (scores 0-6 with higher scores indicating a worse baseline) [4].

On arrival, his examination was non-focal. On presentation, the National Institutes of Health Stroke Scale (NIHSS) (grading score for acute stroke presentations with scores from 0 to 42, with higher scores indicating worse clinical symptoms [5]) was 0. He was found to be in new-onset atrial fibrillation, and the heart rate was well controlled. The patient did not receive IV thrombolytics since his symptoms resolved before he arrived at the hospital.

**Figure 1:**
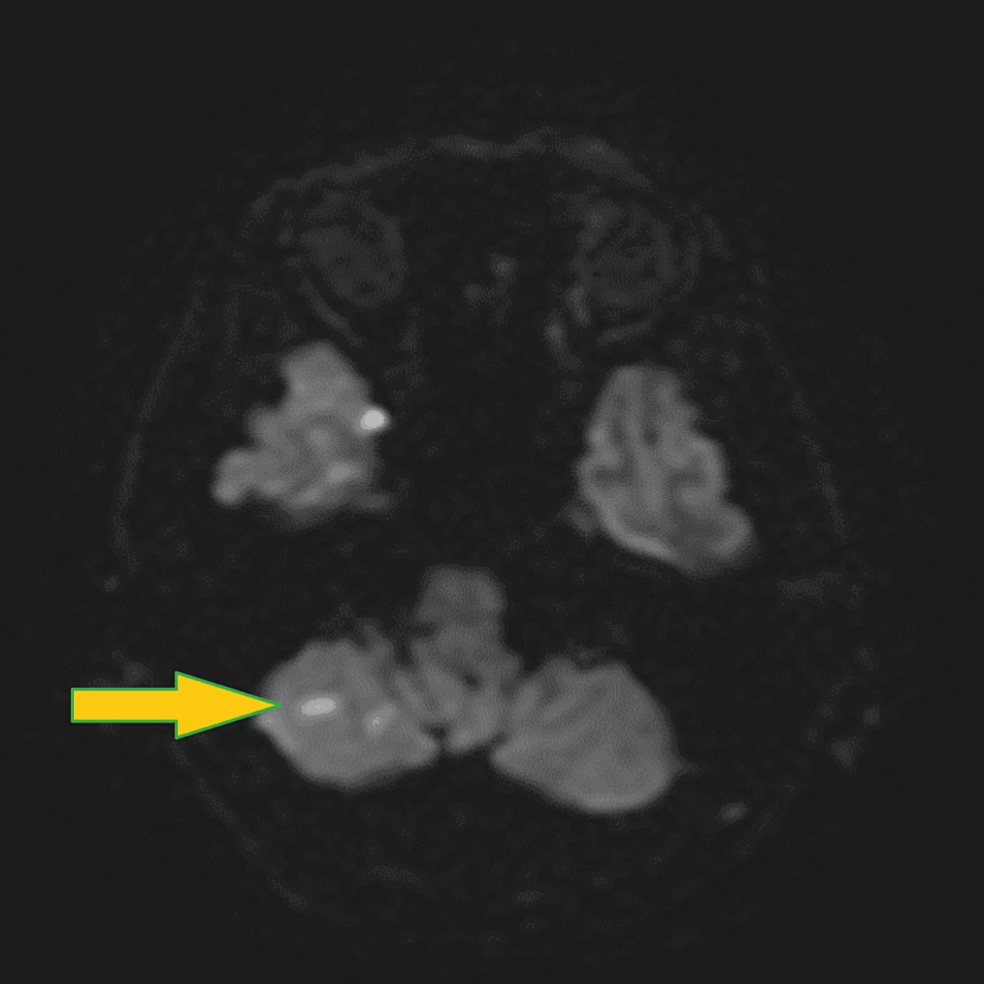
Brain MRI without contrast DWI sequence shows the cerebellar infarcts (arrow). MRI: magnetic resonance imaging, DWI: diffusion-weighted imaging

**Figure 2:**
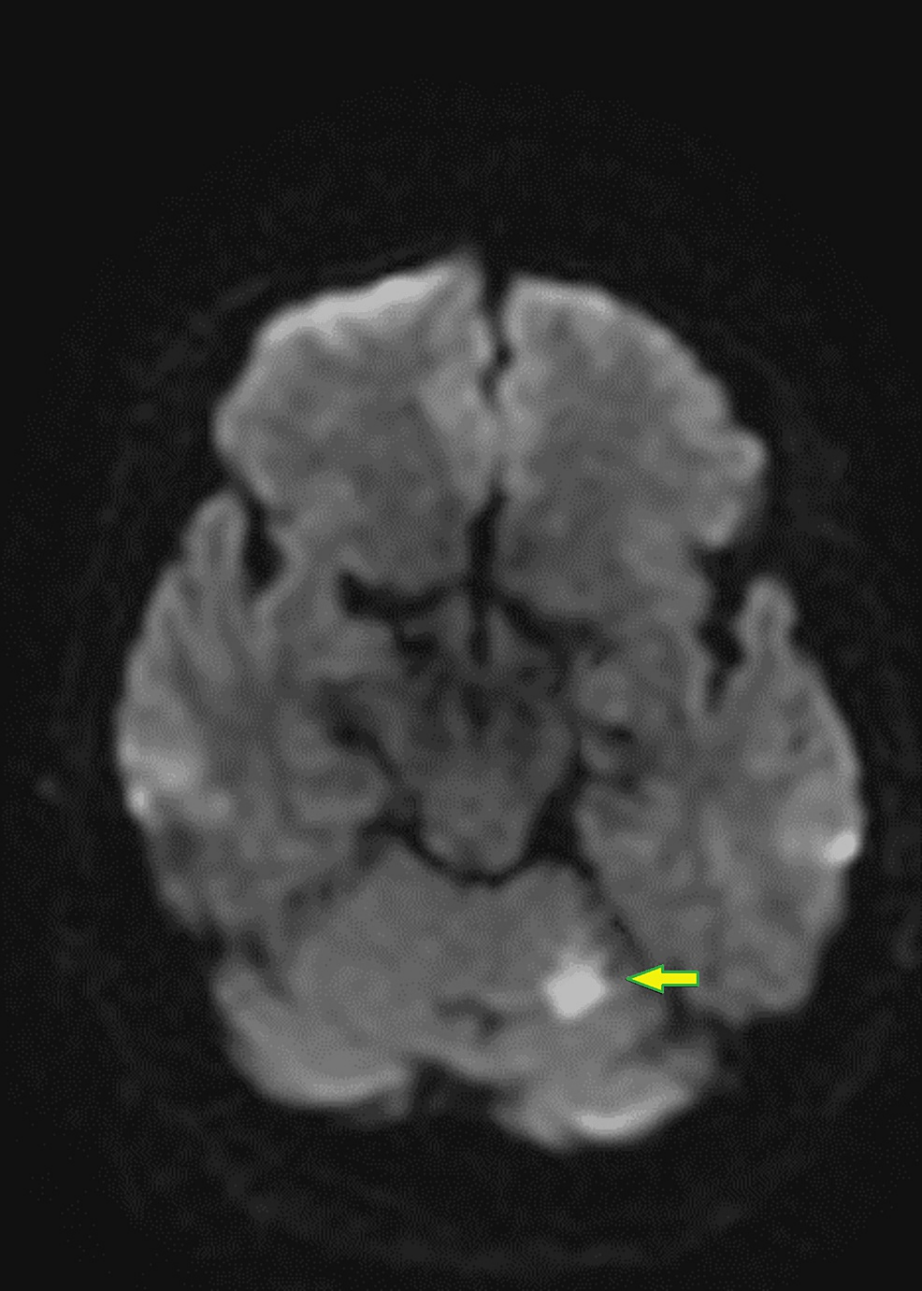
Brain MRI without contrast DWI sequence shows an acute appearing infarct in the left hemi-cerebellar hemisphere (arrow). MRI: magnetic resonance imaging, DWI: diffusion-weighted imaging

Non-contrast head computed tomography (CT) was unremarkable for acute changes. Magnetic resonance imaging (MRI) of the brain without contrast and non-contrast magnetic resonance angiography (MRA) of the head and neck showed bilateral cerebellar ischemic strokes (Figure 1 and Figure 2) associated with focal high-grade stenosis involving the proximal basilar artery.

**Figure 3:**
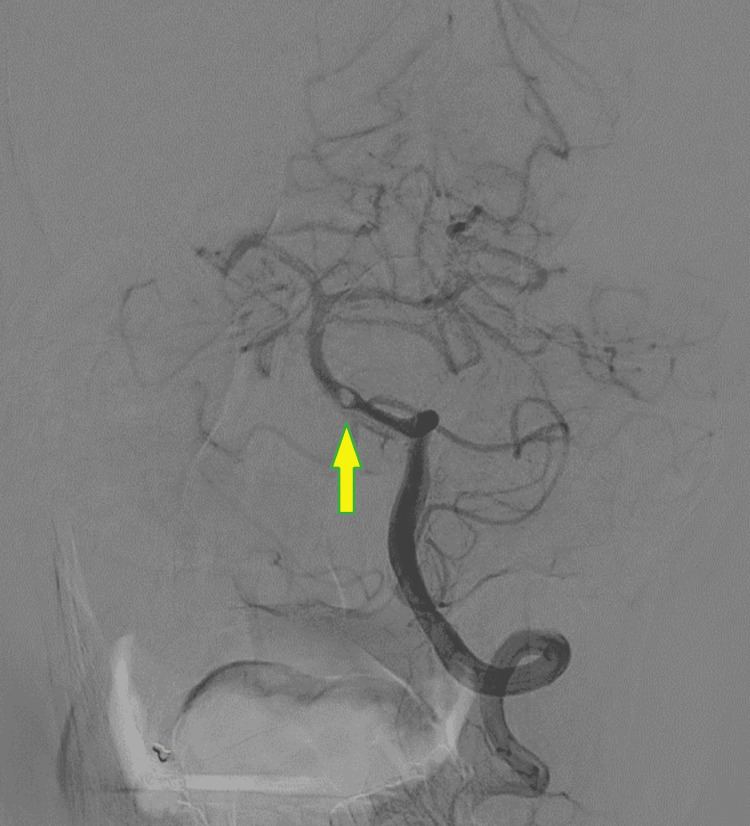
Conventional cerebral angiogram The image shows the subocclusive thrombus with distal flow into the basilar artery and posterior cerebral arteries (arrow).

Transthoracic echo did not show any evidence of cardiac thrombus. The patient underwent a diagnostic cerebral angiogram to evaluate the basilar artery lesion further. On the angiogram, the stenotic lesion appeared as a subocclusive thrombus (Figure 3), and there was no evidence of atherosclerosis of the remainder of the circulation.

After multidisciplinary discussions, initiating anticoagulation with apixaban 5 mg daily was made, given the new onset of atrial fibrillation without any bridging. Rosuvastatin 20 mg daily was added. Tobacco cessation counseling was provided during hospitalization.

Subsequently, the patient had a follow-up CT angiogram of the head and neck in eight weeks, which showed resolution of the subocclusive thrombus. The patient had no interval complaints or focal neurological deficits during the neurology office evaluation 12 weeks after ischemic stroke.

## Discussion

The basilar artery is one of the major vessels supplying the posterior circulation of the brain. It is formed at the pontomedullary junction by the two vertebral arteries joining together. It runs along the ventral surface of the pons and ends as the posterior cerebral arteries. The significant branches originating from the basilar artery are the superior cerebellar artery, internal auditory arteries, and anterior inferior cerebral arteries. It also gives off numerous pontine perforator branches. The basilar artery supplies to the brainstem, cerebellum, thalamus, and occipital and medial temporal lobes [6].

About 20% of ischemic strokes involve posterior circulation. Common etiologies include cardioembolism, significant artery atherosclerosis, or hypercoagulability [6]. The major risk factors include smoking, hypertension, dyslipidemia, and diabetes mellitus type 2. Posterior circulation strokes are challenging due to nonspecific symptoms at presentation. Basilar artery strokes can lead to significant mortality and morbidity, including locked-in syndrome without treatment.

Depending on the occlusion location, patients can present with vertigo, paresthesia, dysarthria, gait imbalance, and visual symptoms. Without adequate history and a high degree of suspicion, the chances of missing a posterior circulation stroke are very high.

Rapid diagnosis of the occlusion can be made with a CT angiogram of the head and neck or a magnetic resonance angiogram of the head and neck. CT perfusion of the brain is an excellent tool to estimate the area of the ischemic core and penumbra in the anterior circulation. However, the test is not reliable in the posterior circulation. Further definitive estimation of the burden of infarction is with diffusion-weighted MRI of the brain. MRI of the brain has its challenges with longer scanning time and contraindications for some patients with foreign bodies.

Stroke workup includes telemetry monitoring, checking lipid panel, hemoglobin A1c, cardiac ultrasound, and neurovascular imaging. Patients are treated either with antiplatelet or anticoagulation based on the cause. Patients typically start on antiplatelets if the etiology is presumed to be small vessel disease without atrial fibrillation. If the patient has a transient stroke symptom or NIHSS score of <3, the patient would be on dual therapy for 21 days, followed by monotherapy [7-9]. The commonly used antiplatelet drugs are aspirin, clopidogrel, ticagrelor, or cilostazol. Anticoagulation, such as apixaban, rivaroxaban, warfarin, or dabigatran, is used in patients with atrial fibrillation. If the patient has 70%-99% stenosis of the basilar artery, the patient should be on dual therapy with aspirin 325 mg daily and Plavix 75 mg daily for 90 days, followed by monotherapy [10]. Adequate control of secondary risk factors such as high-intensity statin for dyslipidemia to target a goal low-density lipoprotein (LDL) of less than 70, appropriate management for diabetes, and smoking cessation should be done in all patients.

Over the last decade, endovascular therapies have significantly impacted the outcomes [11,12] of anterior circulation strokes. Endovascular therapies with posterior circulation strokes are evolving and recently have shown clear benefits [13,14] compared to medical management. The Basilar Artery International Cooperation Study (BASICS) and Endovascular Treatment versus Standard Medical Treatment for Vertebrobasilar Artery Occlusion (BEST) trials did not show a significant use of thrombectomy for basilar strokes at three months [15,16]. Still, they suggested the possibility of better outcomes in patients with moderate to severe symptoms. The Endovascular Treatment for Acute Basilar-Artery Occlusion (ATTENTION) and Basilar Artery Occlusion Chinese Endovascular (BAOCHE) trials showed the definitive advantage of thrombectomy in decreasing disability and mortality, especially in patients with moderate to severe symptoms [17,18]. A recent meta-analysis showed that endovascular thrombectomy for basilar occlusion significantly reduced 90-day mortality [19]. The American Heart Association (AHA) guidelines state that, despite uncertain benefits, endovascular thrombectomy may be reasonable in select basilar artery occlusion within the first six hours of stroke onset (class IIb, level of evidence C).

## Conclusions

Basilar artery strokes can present with various nonspecific symptoms. A very high degree of suspicion and knowledge of the disease process is crucial to early diagnosis; if not identified, basilar strokes can lead to potentially devastating outcomes. Patients can have good outcomes, as described in this case, if identified early and appropriately treated. Endovascular management is a promising treatment option and can be lifesaving in certain patients with basilar artery stroke.
